# Complicated type B aortic dissection in a pregnant woman with Marfan syndrome

**DOI:** 10.1016/j.jvscit.2024.101561

**Published:** 2024-07-02

**Authors:** Mohammad M. Zagzoog, Sean A. Crawford, Jean-Michel Davaine

**Affiliations:** a Department of Vascular and Endovascular Surgery, Pitié-Salpetriere University Hospital, Sorbonne Université, Faculty of Medicine, Paris, France; b Faculty of Medicine, Sorbonne University, Paris, France; c Peter Munk Cardiac Centre, Toronto General Hospital, University Health Network, Toronto, ON, Canada

**Keywords:** Aortic dissection, Retrograde type A aortic dissection, Pregnancy, Marfan syndrome, Thoracic endovascular aortic repair

## Abstract

Marfan syndrome is a rare inherited connective tissue disorder that can result in significant morbidity and mortality. We report a case of a 29-year-old pregnant woman presenting with an acute type B aortic dissection. Owing to cardiopulmonary decompensation and intestinal malperfusion, she underwent an emergency cesarean section followed by left subclavian to carotid transposition and thoracic endovascular aortic repair that was complicated by a retrograde type A aortic dissection and was managed surgically. Molecular testing confirmed the diagnosis of Marfan syndrome. This case highlights that multidisciplinary and hybrid management of challenging cases of acute aortic syndromes can result in a favorable outcome.

Aortic dissection (AD) related to pregnancy is a rare phenomenon and accounts for 0.1% to 0.4% of all ADs and represents 0.0004% of all pregnancies.^1^^,^^2^ Despite its rarity, AD in pregnancy remains one of the leading causes of death in pregnant patients.^3^ Moreover, pregnancy associated with Marfan syndrome (MFS) leaves patients particularly susceptible to AD.^4^, ^5^, ^6^ Because of the rarity and limited clinical experience with acute AD in pregnant patients with MFS, the optimal management strategy is not well-established and remains controversial.^7^ The related knowledge is mainly based on case reports and small series, and thus no robust recommendations are available to date.^8^ In the present report, we describe our experience with a case of a complicated type B aortic dissection (TBAD) treated by a hybrid approach, which further resulted in a retrograde type A aortic dissection (RTAD) in a pregnant patient with MFS. Patient consent was obtained for this case report.

## Case report

**Fig 1:**
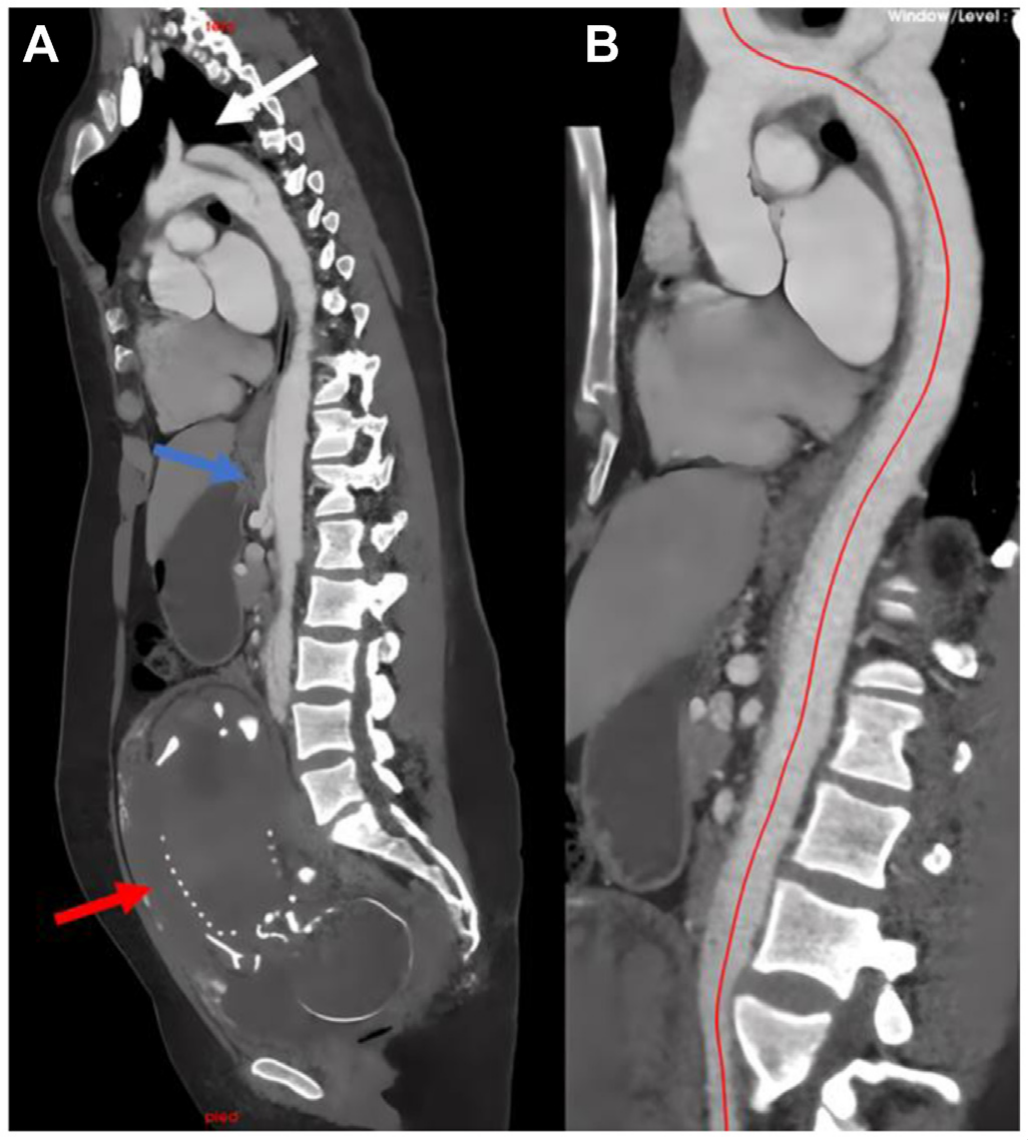
**(A)** Sagittal view from preoperative computed tomography angiography (CTA) showing the fetus (red arrow) and type B aortic dissection (TBAD) with an entry tear (white arrow) located in zone 3 and involving the visceral arteries (bleu arrow). **(B)** Sagittal view with central line demonstrates a bovine aortic arch and TBAD with no evidence of aortic root dilatation, intramural hematoma or aortopathy.

A previously healthy, 29-year-old gravida 2 para 1 woman presented in our department during her 30th week of gestation with the sudden onset of chest pain radiating to the back. She had delivered uneventfully 2 years earlier. On initial presentation, her blood pressure was 150/73 mm Hg. On physical examination, she had the classic clinical manifestations of MFS, including tall limbs, thin appearance, malar hypoplasia, arachnodactyly, joint hypermobility, and ectopia lentis. However, she had no family history of aortic dissection or connective tissue disease. She underwent a low-dose computed tomography angiogram (CTA) that showed a bovine aortic arch and acute TBAD (B_3,10_) with an entry tear located in zone 3 and extending down to both iliac arteries (Fig 1, *A* and *B*) with no evidence of aortic root dilatation, intramural hematoma, or aortopathy. Her laboratory markers were unremarkable. She was admitted to the intensive care unit for medical therapy, including antihypertensive treatment (labetalol, urapidil, and nicardipine). She was hemodynamically stable. On the third day, she developed an episode of severe hypertension unresponsive to antihypertensive treatments and respiratory decompensation requiring oxygen therapy up to 6 L/min using an O_2_ face mask. A diagnosis of a cardiogenic pulmonary edema of undetermined etiology was made with left ventricular systolic function at 50% and left ventricular dilation, no dilation of the right cavities, and an unchanged electrocardiogram with no evidence for coronary artery disease. A multidisciplinary discussion involving vascular surgeons, obstetricians, neonatologists, and anesthesiologists resulted in the decision to undertake an emergency cesarean delivery, which was both successful and uneventful. After the delivery, her blood pressure was well-controlled under the same antihypertensive agents. Eight days later, she developed severe abdominal pain and intestinal malperfusion diagnosed through a CTA. She was taken to the operating room for left subclavian to carotid transposition and thoracic endovascular aortic repair (TEVAR) by using a 26-mm (oversizing 10% based on the CTA images with central line analysis) TAG conformable thoracic stent graft (W. L. Gore & Associates, Flagstaff, AZ) landing in zone 1 (nondissected zone) of the aortic arch. The procedure was carried out uneventfully.

Three days after her aortic procedure, she developed a sudden onset of chest pain. The CTA showed a retrograde aortic dissection, which was treated by ascending aortic replacement with a branched Dacron graft (26 mm) and reimplantation of the innominate artery under cardiopulmonary bypass, partial hypothermic circulatory arrest at 28°C, and perfusing in an anterograde fashion into the left carotid and right subclavian arteries. The postoperative course was uneventful, and she was discharged home after 14 days. Mother and infant continue to do well. She was then on three antihypertensive medications and aspirin therapy.

**Fig 2:**
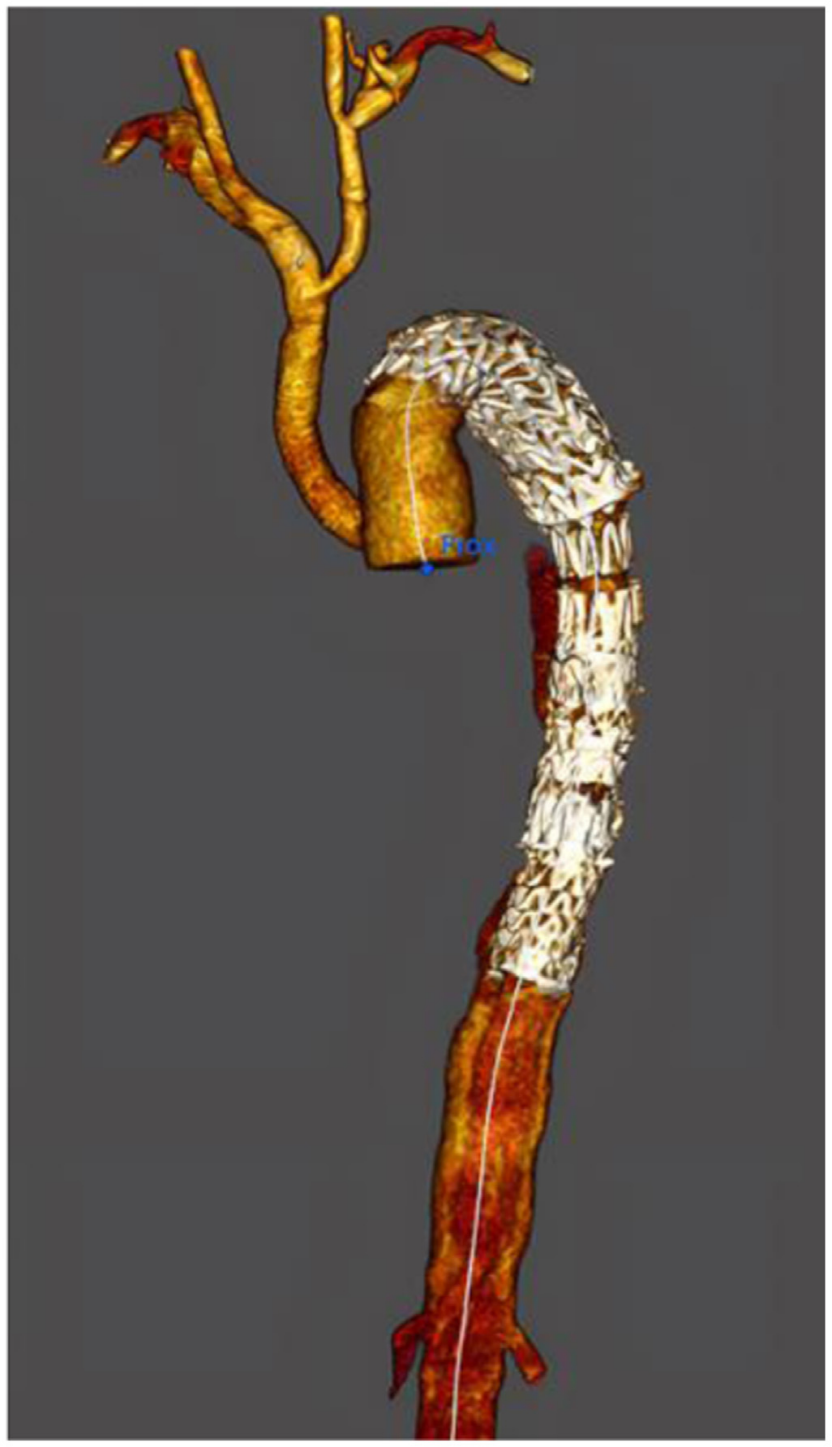
Three-dimensional computed tomography angiogram (CTA) at 6 months of follow-up demonstrating the aortic reconstruction showing the left subclavian to carotid transposition and ascending aortic replacement with reimplantation of the innominate artery by a bypass graft.

Histologic and genetic analyses confirmed the assumption of MFS with a heterozygote de novo mutation of the FBN1 gene caused by a frameshift mutation. After 6 months of follow-up, a CTA (Fig 2) revealed the patency of the aortic graft and the stability of the distal aortic dissection.

## Discussion

According to a US population-based health care database, the incidence of AD during pregnancy and the puerperium has increased from 2002 to 2017 and has been reflected directly by the increase in the incidence of TBAD during pregnancy and the puerperium. More than one-half (58.8%) of these patients presented with AD during the third trimester.^3^ This study found that as many as 21.9% of pregnancy-related ADs were in patients with MFS, and that MFS was associated with an extremely high risk of developing AD during pregnancy and the puerperium. According to international guidelines, stable patients with uncomplicated TBAD should receive optimal medical treatment.^9^ However, the medical management of uncomplicated TBAD still confers a 30-day mortality rate of 10% and a decreased long-term survival rate. Interest remains in determining whether early endovascular intervention might decrease the risk of downstream complications or negative aortic remodeling, particularly in high-risk patients such as the present one.

Ma et al^4^ reported their experience in treating female patients with MFS during pregnancy with TBAD. They recommended that medical therapy for uncomplicated TBAD and complicated TBAD should be managed urgently with a delivery-first strategy, followed by aortic repair.^4^ Saving the fetus is always a worthwhile and often attainable goal. However, aortic repair should be prioritized over delivery for patients with visceral ischemia and signs of contained or impending aortic rupture. In this work, the authors noted that the early fetal survival before 28 gestational weeks was significantly lower compared with that after 28 gestational weeks (*P* < .001).^4^ TEVAR for complicated TBAD has become the first-line treatment owing to its lower morbidity and mortality than conventional open surgical repair.^10^, ^11^, ^12^ The primary goal of TEVAR is true lumen expansion, reducing the pressure inside the false lumen and thrombosis by covering the intimal tear (entry) of the aorta.^13^ However, as illustrated here, in a situation wherein the aortic vessel wall is particularly fragile, TEVAR can trigger a RTAD, a potentially fatal complication, whose incidence ranges from 1.3% to 17.9% and mortality from 33.4% to 42.0%.^14^ In the presented case, the intimal tear presented in zone 3 of the aortic arch and, given the presence of a bovine aortic arch, the decision was made to land the stent graft as proximal as possible in a healthy nondissected aorta just after the innominate artery. Therefore, in our opinion, this strategy increased the risk of RTAD in this case.

The operative strategies for the rescue of these patients from this complication are similar to those for acute type A dissection. Thus, treating these challenging cases in tertiary care centers is critical. Another controversial aspect of this case is the effect of radiation exposure during pregnancy. The fetus is most susceptible to radiation during organogenesis (2-7 weeks of gestation) and in the first trimester, and more resistant during the second and third trimesters.^15^ It is usually considered that the maternal benefit of an early and accurate diagnosis may outweigh the fetal risk of radiation exposure. Coordination with a radiologist is helpful in modifying the CT protocol to decrease the total radiation dose and the use of unnecessary multiphasic protocols. Here, after initial CTA and before child delivery after 48 hours of intensive care unit admission, angio-magnetic resonance imaging was performed at 48 hours to decrease the overall irradiation.

This case highlights the potential morbidity of treating an aortic dissection in pregnant women with MFS. It is critical to identify improved methods to prevent pregnancy-related AD in patients with MFS. The existing literature suggests that women with high-risk pregnancies, such as those with MFS, should be closely monitored by a multidisciplinary team owing to the increased risk of aortic related complications especially in the third trimester, when the risk is highest.^4^^,^^7^^,^^8^ Follow-up of such patients should be realized in close vicinity of tertiary centers if possible.

## Disclosures

None.
